# Sesamoid correction achieved during the learning curve for Scarf-Akin osteotomy without lateral soft-tissue release: a single-centre prospective observational study

**DOI:** 10.1007/s00402-025-05883-z

**Published:** 2025-05-09

**Authors:** Valentina Rossi, Mohammed Hemmati, Paolo Magliulo, Agostino Giordano, Antonio Izzo, Massimo Mariconda, Alessio Bernasconi

**Affiliations:** Trauma and Orthopaedics Unit, Department of Public Health, University of Naples Federico II, Naples, Italy

**Keywords:** Hallux valgus, Scarf, Akin, Sesamoid, Osteotomy

## Abstract

**Introduction:**

Scarf-Akin osteotomy (with or without lateral soft-tissue release (LSTR)) is commonly performed to treat hallux valgus (HV). An insufficient correction of sesamoids can be a risk factor for early recurrence of the condition. We set out to determine 1) the radiographic correction achieved after Scarf-Akin osteotomy performed without LSTR and 2) the degree of correction of sesamoids obtained during the learning curve of the technique.

**Materials and methods:**

In this prospective single-centre study, the first 25 feet (25 patients, mean age 55.2 years, 14 left) undergone Scarf-Akin osteotomy without LSTR by a single foot and ankle orthopaedic consultant in his first year of activity were enrolled and followed-up at 1-year. On weightbearing standard pre-operative and 1-year follow-up radiographs two independent observers (senior residents) assessed and compared the hallux valgus angle (HVA), 1st and 2nd intermetatarsal Angle (IMA), distal metatarsal articular angle (DMAA) and tibial sesamoid position (SP, according to the Hardy and Clapham system). The inter and intraobserver reliability of measurements along with the correlation between the improvement achieved in different parameters and the number of cases performed were tested. Intra and post-operative complications were compared between the early (first 12) and late learning periods.

**Results:**

The inter and intraobserver agreement for the radiographic parameters investigated was excellent in all cases (ICC always > 0.92). A statistically significant improvement in mean HVA (from 36 ± 9.8 to 16.3 ± 2.8 degrees), mean IMA (from 14.5 ± 2.3 to 9.9 ± 1.5 degrees), mean DMAA (from 19.4 ± 4.4 to 11.4 ± 1.9 degrees) and median SP (from 4 (IQR, 3–6) to 2 (IQR, 1–2) points) was demonstrated in the cohort (p < 0.001 in all cases). There was a strong positive significant correlation between the progression of cases over time and the improvement achieved in terms of SP (R = 0.60, p = 0.003). Conversely, no significant correlation was demonstrated when comparing the improvement obtained in HVA, IMA and DMAA with the number of cases performed (p > 0.05 in all cases). One complication occurred during the first 12 cases (1 transfer metatarsalgia) and 1 during the last 13 (1 intra-operative fracture).

**Conclusion:**

In this series, a satisfactory correction of HV after Scarf-Akin osteotomy was obtained without releasing lateral soft-tissues. Beginner surgeons should be aware that restoring sesamoid position may be more challenging as compared to correcting other angles during the first cases.

**Level of evidence:**

Level IV, prospective case series.

## Introduction

The term *hallux valgus* (HV)*,* introduced by Carl Hueter in 1871 to define a static subluxation of the first metatarsophalangeal joint (MTPJ), commonly indicates a lateral deviation of the great toe associated with a medial deviation of the first metatarsal bone, with or without coexisting pronation and subluxation of the first MTPJ [1]. This condition, which is one of the most frequent referrals to foot and ankle specialists, has been linked to functional disability, pain, impaired gait patterns, poor balance and increased fall risk in older adults [2].

Treatment of HV includes both nonoperative and surgical management. Nonoperative treatment may alleviate symptoms but does not correct the deformity. Surgery is considered in patients who fail nonoperative treatment with the goal of pain relief, correction of the deformity, improvement of the first ray stability, and improvement of the quality of life. The most appropriate technique is usually selected from a wide variety of available techniques, also based on the habits of different surgeons [3]. Among these, Scarf osteotomy is an effective technique for HV deformity which has become popular around three decades ago since it provides precise control over metatarsal length, elevation, and position [4, 5].

From a technical standpoint, in a landmark study by Okuda et al. the authors found a significant relationship between the grade of sesamoid displacement and the hallux valgus angle after a proximal metatarsal osteotomy and showed that postoperative incomplete reduction of the sesamoids can be a risk factor for the recurrence of HV [6]. In another study, Seng et al. demonstrated that a learning curve existed for Scarf osteotomy performed along with lateral soft tissue release (LSTR) in order to correct the position of sesamoids [7]. However, to the best of our knowledge, the learning curve of Scarf osteotomy performed without LSTR has never been assessed so far.

With this background, we set out to determine (1) the radiographic correction achieved after Scarf-Akin osteotomy performed without LSTR and (2) the degree of correction of sesamoids obtained during the learning curve of the technique. We hypothesized that a satisfactory correction of HV after Scarf-Akin osteotomy could be obtained without releasing lateral soft-tissues both in terms of angular deformity and sesamoid correction.

## Methods

### Study design

All procedures performed in this prospective single-center study complied with the principles of the Helsinki Declaration and its later amendments or comparable ethical standards. The study followed STROBE (Strengthening the Reporting of Observational Studies in Epidemiology) guidelines and was approved by the local institute relevant Review Board. All patients involved provided a written consent to take part to the study.

### Inclusion criteria

The first 25 feet (25 patients, mean age 55.2 years, 14 left) which undergone Scarf-Akin osteotomy without LSTR by a single foot and ankle orthopaedic consultant in his first year of activity (from June 2020 to June 2021) at the University Federico II of Naples (Naples, Italy) were enrolled and followed-up at 1-year. No patient was lost during such follow-up.

### Data collection

Baseline demographics (age and sex) were stored in a password-protected dedicated database, in which a numeric ID was assigned to each patient. Weightbearing standard pre-operative radiographs were saved as DICOM (Digital Imaging and Communications in Medicine) images. Once they were assigned an ID number as well, they were anonymized. The same protocol was applied to weight-bearing radiographs collected at 1-year of follow-up.

### Measurements

**Fig. 1:**
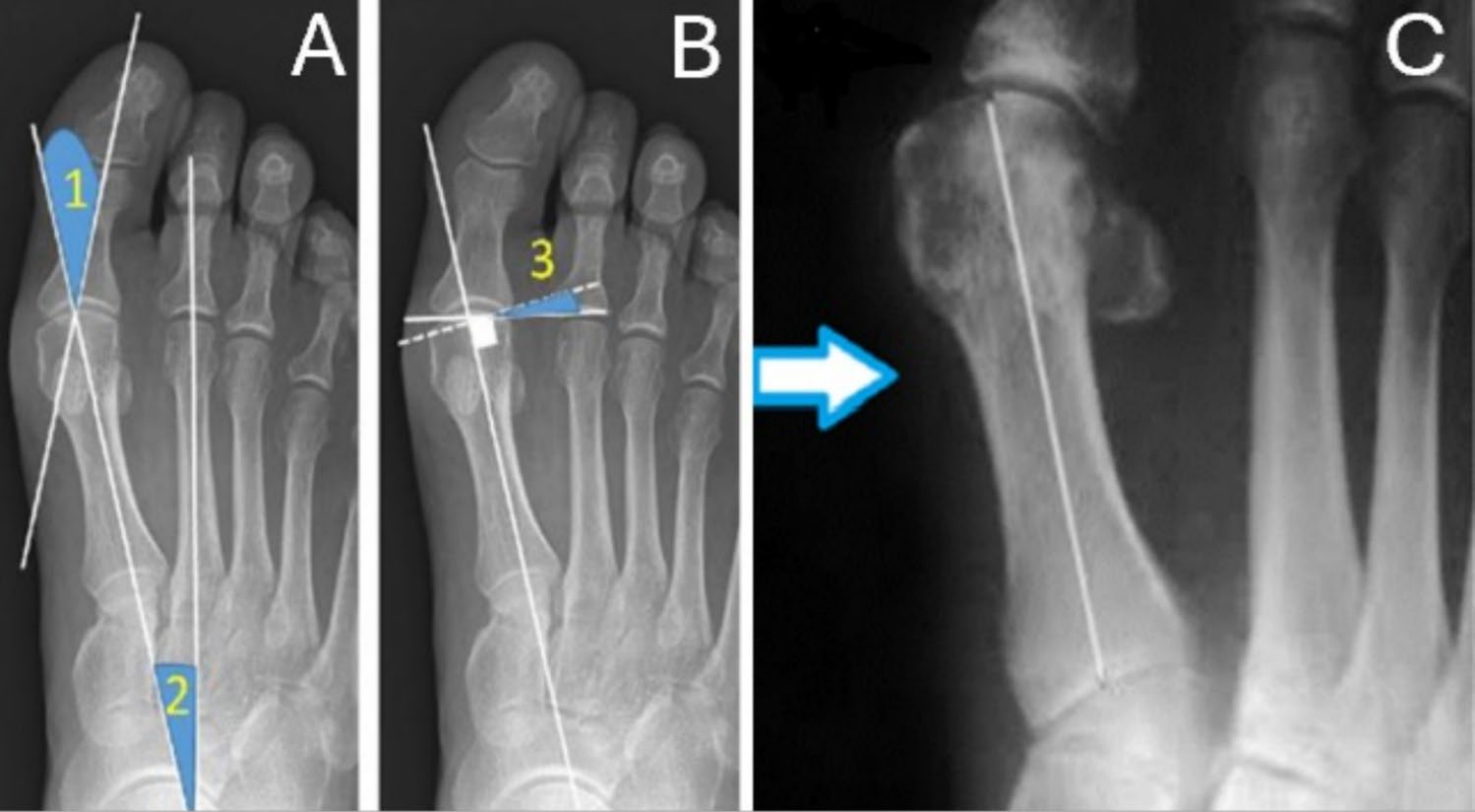
Measurements performed on standard dorsoplantar weightbearing radiographs. **A** 1: hallux valgus angle; 2: intermetatarsal angle; **B** 3: distal metatarsal articular angle; **C** tibial sesamoid position (arrow indicating the tibial sesamoid)

**Fig. 2:**
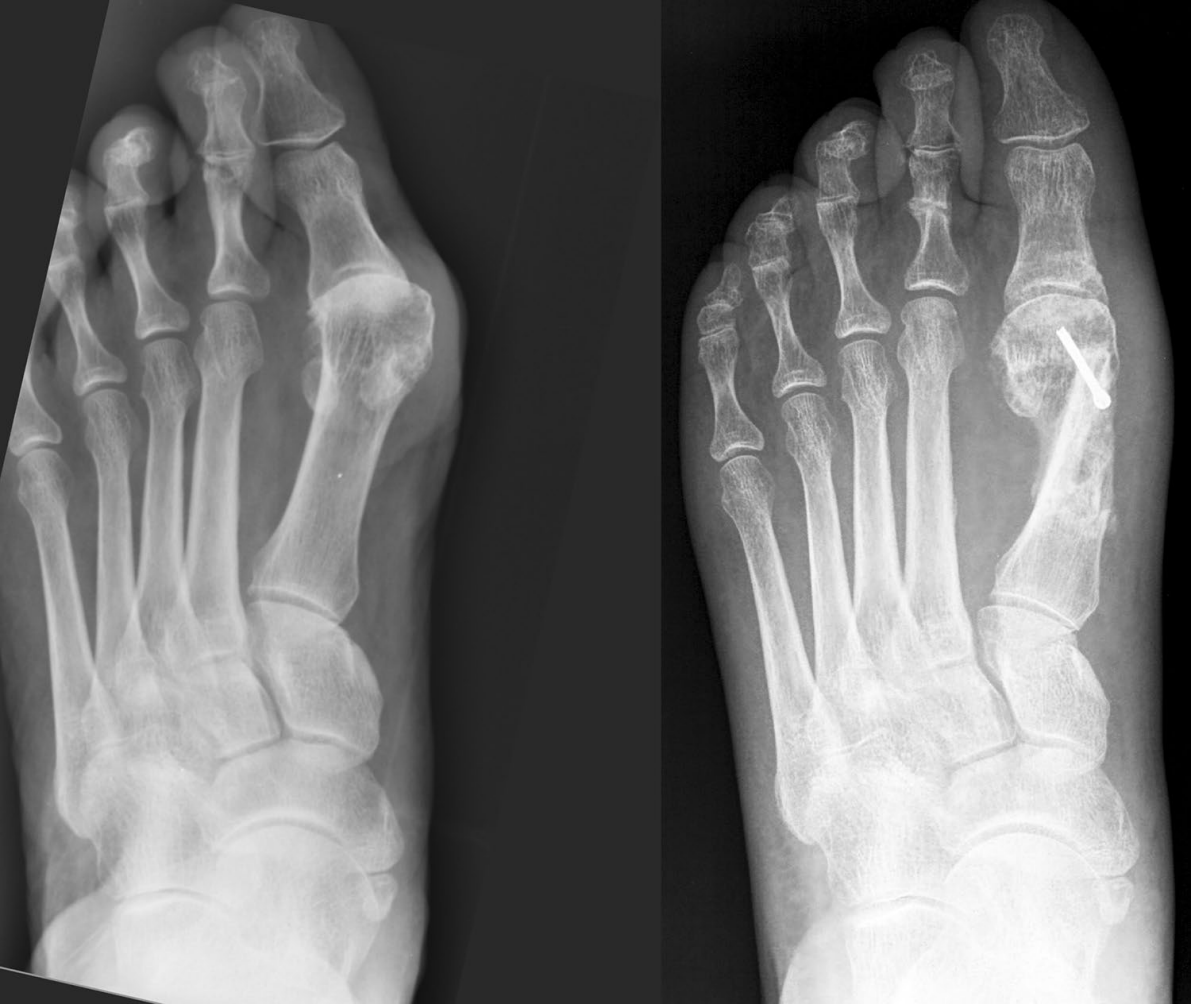
Example of a preoperative (left) and postoperative (right) weightbearing radiographic dorsoplantar view in a patient enrolled in this series. A single screw was used to fix the Scarf osteotomy. Sesamoid position improved from 3 to 1 according to the Hardy and Clapham classification system. No lateral soft tissue release was performed

**Fig. 3:**
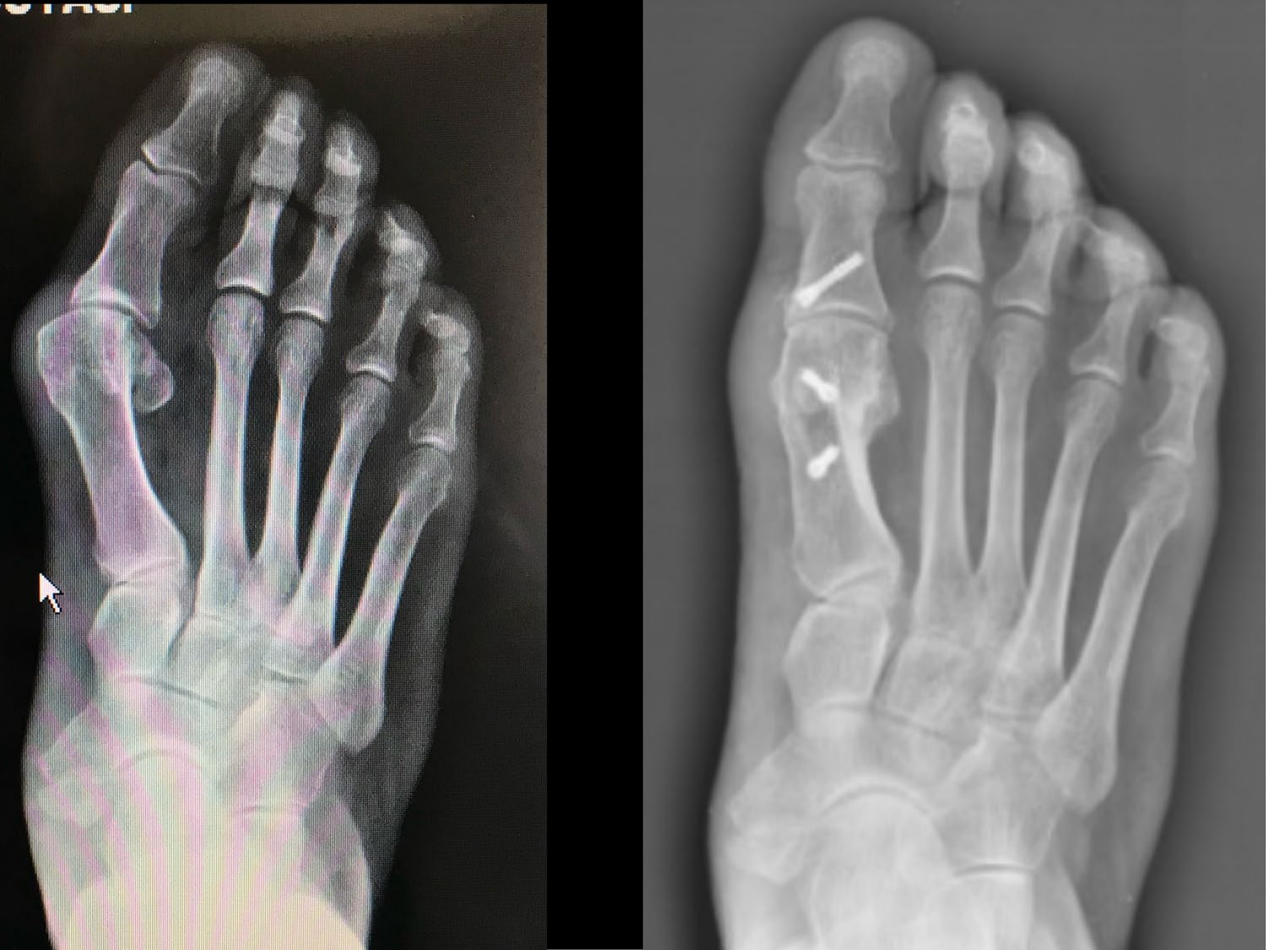
Example of a preoperative (left) and postoperative (right) weightbearing radiographic dorsoplantar view in a patient enrolled in this series. Two screws were used to fix the Scarf osteotomy and one (only case in the series) for the Akin osteotomy. Sesamoid position improved from 6 to 2 according to the Hardy and Clapham classification system. No lateral soft tissue release was performed

On pre-operative and 1-year follow-up radiographs two independent observers (two senior orthopaedic residents (VR and AI)) assessed and compared the hallux valgus angle (HVA), the 1st and 2nd intermetatarsal angle (IMA), the distal metatarsal articular angle (DMAA), and the sesamoid position (SP) (according to the Hardy and Clapham system), as reported in previous literature [7–10] (Figs. 1, 2 and 3). Observers evaluated radiographs in a random order to avoid potential biases related to the learning curve of the technique. The inter and intraobserver reliability for these measurements along with the correlation between the improvement achieved in different parameters and the number of cases performed were tested. Moreover, intra and post-operative complications were compared between the early (first 12) and late learning periods.

### Surgical technique

**Fig. 4:**
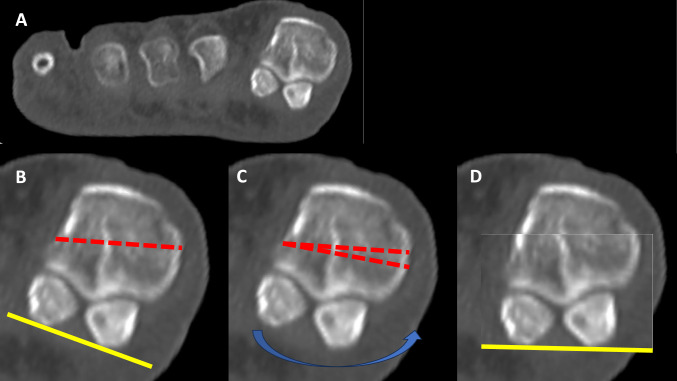
In **A**, coronal computed tomography scan of the metatarsal heads including sesamoids with some degree of pronation. In **B** example of a transverse (longitudinal) cut performed during a Scarf Akin osteotomy. In **C** example of re-cut of the plantar fragment performed in order to remove a wedge and to derotate the first metatarsal head (**D**)

Surgery was performed positioning the patient in a supine position. A tourniquet was positioned above the ankle (third distal leg) and inflated at 250 mmHg. After loco-regional anaesthesia, a medial longitudinal incision was placed medially to the first MTPJ. Capsule was opened and the joint was exposed. The first metatarsal bone was then cut in a Z shape, performing the longitudinal cut first, the transversal distal dorsal cut second and the transversal proximal plantar cut as last. The direction of the transversal cut was decided based on the need to lengthen, shorten or keep the same length of the first metatarsal, which in turn depended on the overall metatarsal parabola and by the clinical scenario (presence or not of metatarsalgia). The correction of pronation was achieved through progressive re-cuts of the longitudinal cut removing small wedges of bone until a satisfactory supination of the metatarsal head was achieved (Fig. 4). The plantar fragment was then shifted laterally and provisionally stabilized using K-wires, then one or two 2.5 mm titanium cannulated screws (AUTOFIX^®^ system, Stryker, Kalamazoo, USA) were positioned for final fixation. An Akin osteotomy was performed to correct the residual interphalangeal valgus and stabilised using external dressings. Only in cases where the lateral hinge was broken and the osteotomy site was unstable a 2.5 mm titanium screw was used in the phalanx as well. Lateral soft tissue release (LSTR) was never performed. After closure, A Mann dressing was applied keeping the hallux hypercorrected in order to close the Akin osteotomy. Patients were allowed to load weight immediately in a forefoot offloading shoe for 4 weeks (or 6 weeks whenever an additional arthrodesis of the interphalangeal joint of the second toe was performed using a temporary K-wire). Normal shoes were recovered after this period. Weightbearing radiographs were taken at 4 weeks, then patients were reviewed at 3, 6 and 12 months.

### Statistical analysis

Data were reported as mean values, standard deviation (SD), and range values (minimum and maximum). Normality of distribution was tested using the Shapiro–Wilk test. The inter and intraobserver reliability for different measurements was tested through the intraclass correlation coefficient (ICC) with 95% confidence intervals (95% CI). Comparison between pre- and post-operative data were performed using Student t-test for normally distributed continuous variables and Wilcoxon rank-sum test for nonnormally-distributed continuous variables. Chi-squared was used for categorical variables. The correlation between variables was assessed using the Pearson correlation coefficient (R). The p value was set at 0.05. Analyses were performed using STATA package.

## Results

The interobserver and intraobserver agreement for the radiographic parameters investigated was excellent in all cases (Table 1).

**Table 1:** Interobserver and intraobserver agreement for radiographic measurements assessed in this cohort

	Interobserver agreement	Intrabserver agreement
	ICC (95% CI)	ICC (95% CI)
Hallux valgus angle	0.99 (95% CI 0.98–0.99)	0.99 (95% CI 0.99–0.99)
Intermetatarsal angle	0.96 (95% CI 0.92–0.98)	0.99 (95% CI 0.99–0.99)
Distal metatarsal articular angle	0.99 (95% CI 0.95–0.99)	0.99 (95% CI 0.98–0.99)
Sesamoid position	0.99 (95% CI 0.95–0.99)	1

*ICC* intraclass correlation coefficient, *95% CI* 95% confidence intervals

**Fig. 5:**
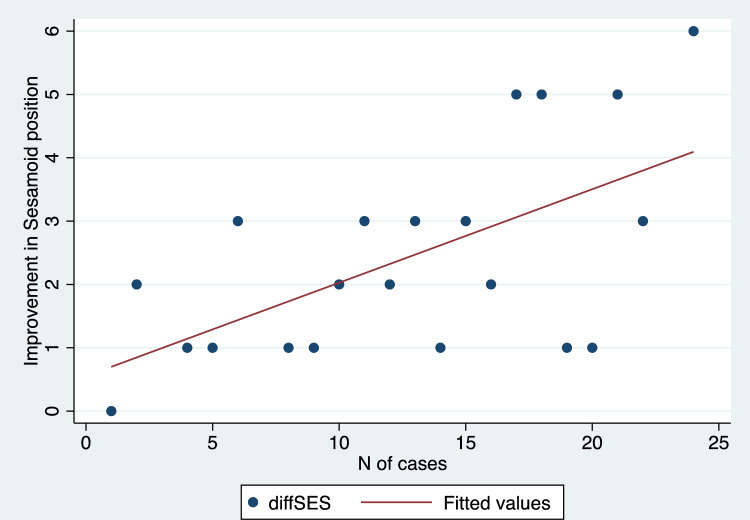
Scatter plot illustrating the correlation between the correction achieved in sesamoid position and the number of cases performed

**Fig. 6:**
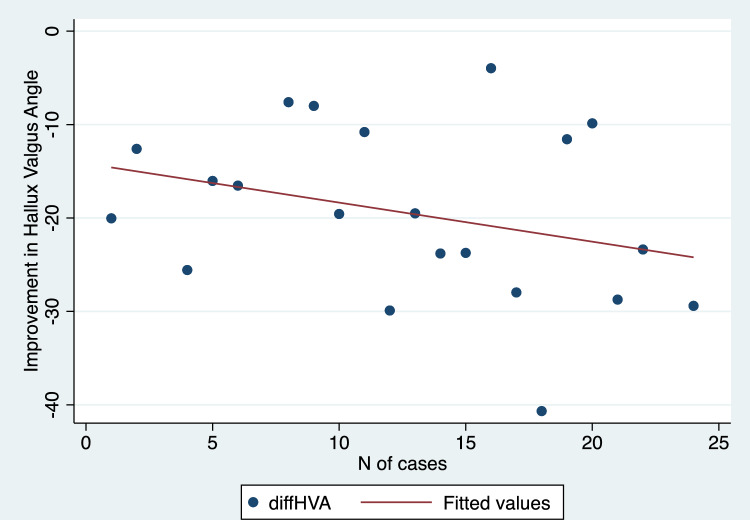
Scatter plot illustrating the correlation between the correction of the hallux valgus angle and the number of cases performed

**Fig. 7:**
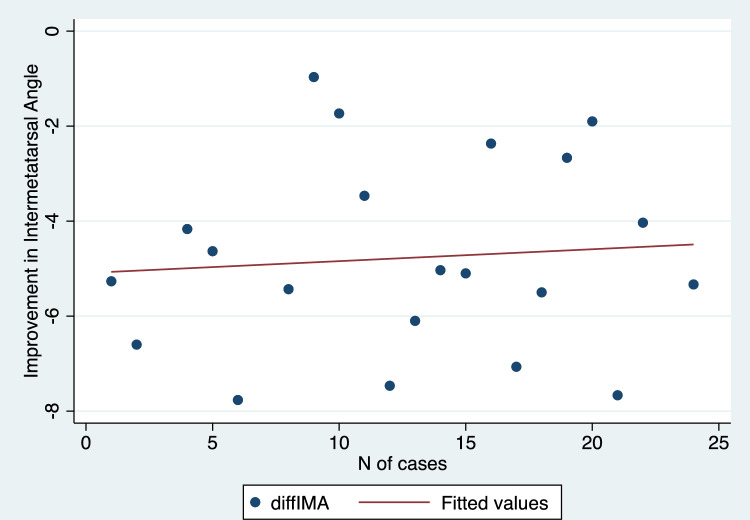
Scatter plot illustrating the correlation between the correction of the 1st and 2nd intermetatarsal angle and the number of cases performed

**Fig. 8:**
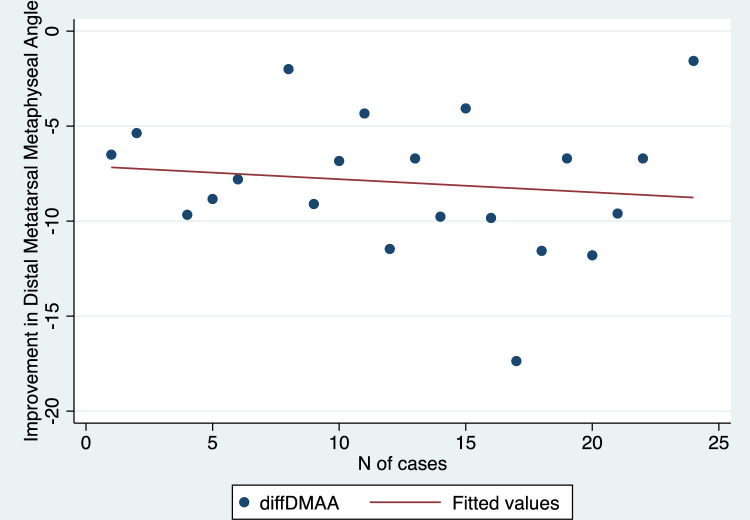
Scatter plot illustrating the correlation between the correction of the distal metatarsal articular angle and the number of cases performed

Mean values with standard deviations and range values for the radiographic parameters assessed in this study have been depicted in Table 2. We found a statistically significant improvement in HVA, IMA, DMAA, and SP comparing preoperative and 1-year follow-up values (p values < 0.001 in all cases) (Table 2). Additionally, there was a strong positive and significant correlation between the progression of cases over time and the improvement achieved in terms of sesamoid position (R = 0.60, p = 0.003) (Fig. 5). Conversely, no significant correlation was demonstrated when comparing the improvement obtained in HVA (R = 0.30, p = 0.17) (Fig. 6), IMA (R = − 0.08, p = 0.71) (Fig. 7), and DMAA (R = 0.12, p = 0.57) (Fig. 8) with the progression of cases.

**Table 2:** Pre- and post-operative values for radiographic measurements assessed in this cohort

	Pre-operative			Post-operative			p value
	Mean	SD	Range	Mean	SD	Range	
Hallux valgus angle (degrees)	36	9.8	20.7–56.3	16.3	4.8	8.2–27	< 0.001*
Intermetatarsal angle (degrees)	14.5	2.3	8.9–19	9.9	1.5	7.1–12.6	< 0.001*
Distal metatarsal articular angle (degrees)	19.4	4.4	12–28.8	11.4	1.9	7.8–15.9	< 0.001*
	Median	IQR	Range	Median	IQR	Range	p value
Sesamoid position (points)	4	3–6	2–7	2	1–2	1–3	< 0.001**

	Median	IQR	Range	Median	IQR	Range	p value
Sesamoid position (points)	4	3–6	2–7	2	1–2	1–3	< 0.001**

*SD* standard deviation, *IQR* interquartile range

*Student t test

**Wilcoxon rank-sum test

In terms of baseline demographics (age: p = 0.07; side: p = 0.68) and pre-operative measurements (HVA: p = 0.6; IMA: p = 0.37; DMAA: p = 0.07, SP: p = 0.11), the first 12 cases were comparable to the last 13 (Table 3). One complication occurred during the first 12 cases (1 transfer metatarsalgia) and 1 during the last 13 (1 intra-operative fracture).

**Table 3:** Baseline demographics and pre-operative radiographic measurements in the first 12 cases and in the last 13

	First 12 cases		Last 13 cases		p value
	Mean	SD	Mean	SD	
Age (y)	55.2	17.1	60.2	6.6	0.07
Preop hallux valgus angle (degrees)	32.5	8.9	39.2	9.8	0.6
Preop intermetatarsal angle (degrees)	14.4	3	14.7	1.6	0.37
Preop distal metatarsal articular angle (degrees)	18	3.5	20.8	4.8	0.07

	%		%		p value
Sex (% of F)	0.65	–	0.74	–	0.68

	Median	IQR	Median	IQR	p value
Preop Sesamoid position (points)	3	3–4	4	3–6	0.11

*SD* standard deviation, *IQR* interquartile range

## Discussion

In this study, while we confirmed that Scarf-Akin osteotomy allows to correct satisfactorily hallux valgus deformity even without the release of lateral soft tissues (as demonstrated by traditional angular measurements like HVA, IMA and DMAA), we also found that the ability to restore an appropriate sesamoid position significantly improves during the first 25 cases. To the best of our knowledge, this is the first study to assess the learning curve of Scarf/Akin osteotomy without LSTR and to suggest that particular attention should be paid to the correction of sesamoids for less experienced surgeons during their first cases. While the technique performed has been the same throughout the whole series reported here, and no clear factor could be identified as responsible for the improved outcome achieved over time, we believe that a progressively increasing experience might have played a crucial role in performing more accurately and efficiently the multiple gestures required in a Scarf-Akin osteotomy.

Upon review of the literature, a great enthusiasm can be remarked around percutaneous procedures to treat HV [11]. Interestingly, in a recent systematic review Ferreira et al. compared the open Scarf/Akin technique and the percutaneous Chevron/Akin (PECA), reporting similar outcomes in terms of radiographic correction, pain and function at 6 months of follow-up, but with a longer radiation exposure time for the PECA [12]. While there is a number of studies reporting the learning curve of percutaneous and/or minimally-invasive techniques [13–19], only a few papers have been published dealing with traditional open techniques [5, 7, 20, 21]. In 2003, Smith et al. analyzed their first 100 cases and reported an overall complication rate of 6%, underlining that these occurred within the first 30 cases [20]. In another study by Samaras et al., the authors reported on 78 HVs treated by Scarf osteotomy followed up at 24 months. While the clinical outcome (AOFAS score) was satisfactory since the very first cases (69 feet were satisfied), they documented a non-negligible 19% complication rate in the whole series. It should be remarked that these authors focused specifically on complications recorded during the learning curve rather than on the efficacy of correction achieved from a radiographic standpoint, which instead was the main target of our analysis, and which represents a strength of our study.

There is agreement in literature that restoring sesamoid position improves the outcome and reduces the recurrence rate after HV surgery [22–25]. In a prospective cohort study by Veracruz-Galvez et al. on patients undergoing Scarf osteotomy, normal sesamoid position (assessed on weightbearing radiographs) was associated with lower pain and better patient satisfaction at two years of follow-up as compared to patients with a residual malalignment [26]. In a 2015 landmark paper, Seng et al. assessed 71 consecutive cases (71 feet) of Scarf osteotomy performed on female patients during a 2.5-year period by the same surgeon. The cases were divided into 3 groups according to the date of surgery, with the first 24 cases assigned to group 1, the next 24 to group 2, and the last 23 to group 3. The median sesamoid position (ranging from 1 to 7 as per the Hardy and Clapham classification system) of the 3 groups at 6 weeks from surgery and patient satisfaction at 6 months were recorded. Since the sesamoid correction was significantly better for the second and third groups than for the first, the authors concluded that in Scarf osteotomy a learning curve of 24 cases was necessary to optimally restore the position of the sesamoid, with a direct effect on reducing the risk of recurrence of the deformity [7]. Looking at our results and bearing in mind Seng’s paper at least two considerations have to be made: first, while our cohort was smaller as compared to their study, we identified a statistically significant linear improvement in the ability to correct sesamoids already during the first 25 cases, demonstrating that in our cohort after 15 cases sesamoid position can be improved by up to 5 grades (as per Hardy and Clapham classification) through surgery. Second, in our series LSTR was never performed (as opposed to Seng’s series) which corroborates the concept that bony cuts might be sufficient to realign sesamoids and that additional gestures on soft tissues may not be necessary (as claimed in previous other studies) [27]. As a matter of fact, the role of LSTR for the correction of TSP has been debated over time [28]. In a meta-analysis of 6 comparative studies (425 patients) assessing open isolated osteotomies vs osteotomies with LSTR the authors concluded that transecting the lateral sesamoid-metatarsal ligament might be beneficial in HV surgery and that the release of the adductor hallucis tendon could be considered in moderate to severe deformities [28]. In another review focusing on percutaneous techniques, Izzo et al. found no evidence that LSTR reduced the risk of recurrence at a mean 4-year follow-up nor improved the clinical and radiological outcome after surgery [29]. It’s also true that in some other studies the LSTR is performed ‘only if needed’, which is common to multiple surgeons but which makes difficult to generalize results [30]. Only a specific randomized comparative analysis taking into account all possible covariations involved in HV correction (i.e., surgeon-related factors and patient-related factors) will enable to draw a clearer conclusion on the value of LSTR in this setting.

The authors acknowledge some limitations for the current study. First, the limited sample size and the absence of a statistical power analysis. Second, the short follow-up at 1-year did not allow to assess properly the recurrence rate in this cohort. However, since our target was to assess the immediate radiographic correction, we believe that this should not have biases our findings and conclusions. Third, the use of radiographs to assess of angles may affect our results due to inherent limitations of bi-dimensional imaging. With this in mind, while we are aware that weight-bearing compute tomography might have helped to overcome this issue, such technology was not available at our institution at the time of the study. In addition, sesamoid-view radiographs, which are not part of our routine protocol for HV patients, might have helped to correctly determine the position of sesamoids before and after surgery. Fourth, considering the anatomical role of the lateral suspensory ligament, which according to some papers could be retracted in long-standing hallux valgus [27], we believe that a comparative design would have been desirable to evaluate the correlation between LSTR and the correction of sesamoids, which, as overmentioned, to the best of our knowledge has never been assessed. Fifth, the cohort included in this study was treated by a surgeon in his first year of practice, which may limit its reproducibility for more senior surgeons. However, this was done on purpose in order to analyse different aspects of the learning curve for Scarf-Akin osteotomy. Last, from a statistical standpoint, it could be argued that sesamoid position may be seen as a categorical variable, and that Spearman’s rank correlation would be more appropriate than Pearson’s correlation coefficient to assess the relationship with other variables. Nevertheless, we did assume that different values effectively represented the position of the bone as a continuum, thus we deliberately chose to use Pearson’s coefficient to run the analysis.

## Conclusion

In this cohort including the first 25 HV patients undergone Scarf-Akin osteotomy by a single foot and ankle surgeon, restoring sesamoid position was more challenging as compared to correcting other angles, which especially less experienced surgeons should be aware of. Overall, a satisfactory correction of the deformity was achievable even without releasing lateral soft tissues.

## Data Availability

Data from this study are stored in the official research database of the Orthopaedic and Trauma Unit of our institution.
